# Performance of Earth Plasters with Graphene-Based Additive

**DOI:** 10.3390/ma17102356

**Published:** 2024-05-15

**Authors:** Paola Gallo Stampino, Letizia Ceccarelli, Marco Caruso, Laura Mascheretti, Giovanni Dotelli, Sergio Sabbadini

**Affiliations:** 1 Department of Chemistry, Materials and Chemical Engineering “G. Natta”, Politecnico di Milano, Piazza Leonardo da Vinci 32, 20133 Milan, Italy; letizia.ceccarelli@polimi.it (L.C.); laura.mascheretti@mail.polimi.it (L.M.); giovanni.dotelli@polimi.it (G.D.); 2 Materials Testing Laboratory, Politecnico di Milano, Via Celoria 3, 20133 Milan, Italy; marco.caruso@polimi.it; 3 Disstudio, Via Piolti de Bianchi 48, 20129 Milan, Italy; s.sabbadini@disstudio.it

**Keywords:** raw-earth plasters, graphene-based additive, adhesion strength

## Abstract

A central debate is the improvement in the mechanical and water resistance of sustainable earthen architecture without additives or stabilizers. This innovative work aims to test the effects of a graphene-based additive, optimized for the improvement in concrete properties, on the strength and water resistance of raw-earth plasters without any stabilizer other than sand. Given the heterogeneous nature of raw earth, three different soils were tested by adding three increasing graphene-based additive contents (0.01, 0.05 and 0.1 wt% of the earth–sand proportion). The link between soil intrinsic properties, i.e., geotechnical and mineralogical properties, and their interaction with the additive were investigated through geotechnical characterization, as well as mineralogical characterization, by XRD and ATR-FTIR analyses. The experimental tests carried out focused on the adhesion properties of the twelve different plasters on standard hollow bricks and on their interaction with water through capillary rise tests and erosion resistance tests. Conclusion from the experimental tests suggests that the graphene-based additive in earth plasters, by increasing the cohesion of the mixture, improves their adhesion performance.

## 1. Introduction

The building industry is globally responsible for a significant portion of CO_2_ emissions [1], so new, more sustainable materials, raw earths among them, are studied to partially substitute concrete-based materials [2]. The main strength of earth-based buildings is the exploitation of local materials, reducing almost completely transport emissions, since raw earths are mixed clays that often come from the excavation site for the foundations of the building itself. Moreover, raw-earth products can be easily disposed of at the end of life, as they can go directly back into the environment when a building is dismantled. Earth-based mortars are also used for wall plastering, since they can provide great advantages in terms of the regulation of the internal climate of the building. Thanks to their high porosity and thermal inertia, they decrease the thermal conductivity of the walls, allowing for a cooler environment in dry and hot climates and delaying the decrease in temperatures [3,4]. This climatic regulation means not only lower energy consumption in the in-use phase of the building but also a healthier indoor environment, as they can also absorb pollutants, enhance air quality [5], and improve acoustic and electromagnetic insulation [6].

The main issue with raw-earth plasters, however, is their low resistance to rainfall and water, which could cause erosion and washout, which is a problem especially for external surfaces and which certainly limits the climatic regions where such buildings could be built.

For this reason, even though plastering applications do not require extreme load-bearing performance, research as to which fibers and additives can be included in mortars to increase their water resistance and overall durability and thus overcome these drawbacks is ongoing. The main drawbacks lie in their lower mechanical resistance and easy washout with water; to improve these properties, a wide variety of additives have been tested, including natural fibers and mineral stabilizers, namely, Portland cement and lime, while the newest trend involves adding very small quantities of innovative nanomaterials [7,8,9]. Among them, graphene is the main one, and it is interesting to find out its effects on different clays present in earth mixes.

Graphene has already been extensively tested as an additive in numerous fields, mostly as a lubricant additive thanks to the easy sliding between the different planes [10], but also as an additive in cement paste for concrete property enhancement [11,12]. Graphene, in the form of nanoplatelets, has proven successful in reducing the CSH gel porosity of cement paste, thus increasing the mechanical performance of the final material [9,13]. Moreover, adding graphene to the mix can provide the material with new properties, making it electrically and thermally conductive [14].

Graphene in raw-earth plasters has been added to decrease the amount of cement stabilizers in raw-earth products. Ngo et al. showed that by adding even small quantities of graphene oxide and graphene (0.01 wt%), the quantity of cement needed to stabilize the raw earth decreases by as much as 5%, and the overall impacts also decrease [8]. Adding graphene to a cement-stabilized earth mix also increases the compactability of the mixture, increasing the mechanical properties (in terms of stiffness and compressive strength) and decreasing its plasticity [14,15].

Of course, the addition of graphene would be responsible for environmental impact increase; for this reason, it is interesting to note that some studies already show the possibility of synthesizing graphene from animal fats or biomass [16,17].

Given the imperative for sustainable materials, current graphene research is focused on developing environmentally friendly sources and synthesis methods. Saha et al. have highlighted the promising performance of graphene-like nanomaterials derived from waste biomass or produced by using natural plant extracts [16].

In this paper, different earth plasters are tested in their adhesion performance and water resistance properties to understand when and how the graphene-based additive can be beneficial. The additive used in this study is a water dispersion with patented composition, optimized to improve concrete mix properties, provided by GrapheneUP^®^ (Prague, Czech Republic). At present, this very innovative research is investigating mixtures with only earth, sand and the additive. 

## 2. Materials and Methods

The earth plasters presented in this paper are sand and earth mixtures containing different graphene-based additive contents (0.01 wt%, 0.05 wt% and 0.1 wt% of total solid mass) and water to ensure workability. The reference mix for each plaster, which is the optimal ratio of earth and sand, is based the composition of the different soils, each of them differing from the other in clay mineral content, as reported in Table 1. The three soils were extracted in three different geographical areas in Italy: the ABS and TC soils come from different parts of the Piedmont region, while T2 comes from the Veneto region. The sand comes from the riverbed of the river Piave in Italy and was washed and sieved through a 2 mm sieve before mixing. The graphene-based additive is an aqueous dispersion of graphene (1.5 wt%) with a patented production process and composition provided by GrapheneUP^®^, who developed this additive to optimize concrete properties. Therefore, the soils in this article are identified by their name (i.e., ABS, T2 and TC), while the plaster mixture, containing sand, is referred to as mix (i.e., ABS-mix, etc.). Each earth plaster containing graphene-based additive is simply labelled with the soil name followed by the graphene-based additive content (i.e., ABS-0.1 G% etc.).

### 2.1. Experimental Procedures

#### 2.1.1. Plaster Mix Preparation

The earth-to-sand ratio was chosen from previous in situ tests that showed the optimal mixtures to ensure no cracking during drying. ABS-mix and TC-mix were prepared in an earth-to-sand ratio of 1:3, while T2-mix in a 1:4 ratio. Each sample was a 5.81 cm diameter disk of 1.5 cm in thickness, cast in a round formwork lined with grease to ensure correct removal of the cast without loss of material before the test. To prepare the reference plaster samples, the earth and sand mixture was prepared and homogenized with a mechanical mixer where deionized water can be added with the aid of a syringe. For the samples containing graphene-based additive, GUP Admixture^®^, after being thoroughly shaken to avoid any aggregation, was added to the earth and sand mixture after initial mixing with a small amount of water. The remaining deionized water was then added to obtain the correct consistency. The content of water to ensure comparable workability across different mixtures was determined through a slump test; normally, this value is around 1/5 by weight of the solid portion of the plaster [6]. To determine the best water content, shrinkage tests were carried out in previous works and were used here as a reference (value of around 18%) [18]. The samples were left to dry until the humidity of the control sample measured with a hygrometer was below 1%.

#### 2.1.2. Earth and Mix Characterization 

The crystalline phases contained in the different soil samples and plaster mixes were determined through XRD analysis with a Bruker D8 Advance diffractometer by using a graphite monochromator and Cu-Kα radiation. After grinding the samples in an agate mortar, a complete analysis was carried out with a scan step of 0.02°, at the 2θ angles between 2° and 70°, and 10.5 s residence time. These tests were carried out with 10 wt% of ZnO to obtain quantitative data on the phases and the amorphous content. Diffractogram quantification was performed through Rietveld refinement with the open-source software Profex 5.2.9 [19,20]. To better define the interstratified clay minerals, oriented and glycolate samples were analyzed between 2° and 30° 2θ, with a step of 0.02 degrees and a residence time of 4 s. 

Further analysis of all the samples was carried out by infrared spectroscopy to identify the main functional groups and to assess the change in the mixtures with the increase in graphene-based additive content. The analysis of absorbance was conducted through the Smart itX diamond accessory for Attenuated Total Reflection (ATR) spectra mounted on a Nicolet iS20 FTIR Spectrometer, with a Nicolet iZ10 spectrometer module by ThermoFisher Scientific (Waltham, MA, USA). Spectra were recorded at room temperature in the range between 500 and 4500 cm^–1^ absorbance, and the identification and interpretation were performed by OMNIC™ Specta Software (https://www.thermofisher.cn/order/catalog/product/cn/en/833-036900, accessed on 1 April 2024).

#### 2.1.3. Geotechnical Characterization

To differentiate among the three soils, the main geotechnical reference properties were also determined. A granulometric curve was determined through *sedimentation* and *sieving* as reported in the EN ISO 17892 standard [21,22], as well as specific gravity and Atterberg limits, and finally, the shrinkage limit was determined through the NF P 94-060-2 French standard [23]. Hygroscopic water content (*g*/*g*) was also determined by the oven-drying method.

#### 2.1.4. Adhesion Resistance

To assess the adhesion performance of each plaster, an adhesion test on bricks was carried out according to the guidelines for raw-earth plasters provided by AsTerre (Petit-Couronne, France) [24]. Four samples of each mixture were cast on the surface to be tested. Before casting the mixtures, a slip (a liquid mixture of soil and water) was brushed on the surfaces to ensure a better interaction with the plaster and thereby improving adhesion. Once the samples were dry, the surfaces onto which they were cast were fixed on a vertical metal grid by means of clamps, and an empty container was secured to the sample with a harness and connected to a load cell to continuously measure the load applied on the sample. The container was progressively filled with sand until its weight was enough to detach the sample from the surface. The load cell registered the maximum load sustained (*F_max_* [N]), which can be translated as a value of maximum stress sustained by measuring the adhesion area (*A_sample_* [mm^2^]). This value of maximum stress is defined as adhesion resistance [MPa] = *F_max_*/*A_sample_*.

#### 2.1.5. Scanning Electron Microscopy 

Morphological surface characterization was carried out with the Scanning Electron Microscope Zeiss EVO 50 EP (Zeiss, Jena, Germany) with the spectrometer OXFORD INCA energy 2000 (Oxford Instruments, Abingdon, UK). The SEM was operated at an electron high tension (EHT) voltage of about 20 kV under high vacuum (around 10−5 torr). The SEM was equipped with different probes to analyze both the composition and the morphology of the samples.

#### 2.1.6. Capillary Rise

The procedure is based on the standard proposed by EN 15801: 2009 [25] adapted for earth mortars to prevent loss of sample due to water erosion, as different methods are proposed in the literature [26,27]. The samples, weighed before the beginning of the test, were positioned on top of saturated porous stone disks immersed in water, making sure that the water was not touching the samples themselves (to prevent material loss by means of erosion). The weight of the porous disk and samples were taken at regular intervals to correlate the change in weight, due to the absorption of water, to the elapsed time. With these data, sorption curves were plotted as water absorbed per unit area (kg/m^2^) versus square root of time (*s*^1/2^); such curves should have an initial linear trend, whose angular coefficient is the capillary coefficient (CC) and should flatten once the maximum amount of water is absorbed and saturation reached [28].

#### 2.1.7. Drop Test for Surface Erosion

The drop test experimental set up consisted of a rain chamber with an autonomous moving table, controlled with a Fishino Shark Arduino board, attached to a hydraulic system that supplied demineralized water from a tank at the selected rate through an array of 26 × 45 27 G capillary needles, with a diameter of 0.4 mm. The movement of the tray was programmed through a FishIDE code which allows one to choose the number of steps and cycles the tray must go through before stopping the test. 

The rate of water supply was regulated through a pump, whose speed was 20 RPM. The test was carried out on the same samples as for the adhesion tests positioned horizontally on a tray, to collect the material at the end of the test. It is important to highlight that the test creates quite a harsh condition, since the horizontal placing of the sample does not allow for any water washout. The reference samples were tested in a preliminary test for 45 min, on the basis of a previous work, proving that 30 min was enough to deplete their stability. Then, all the samples were tested for 4 h to compare the results. To quantify the action of the drop test at the end of it, the samples that kept their structural stability were oven-dried again to measure the amount of material lost.

## 3. Results

The complete geotechnical characterization of the soils can be summarized through the parameters indicated in Table 2. 

The mineralogical composition of the plasters is summarized in Table 3.

**Figure 1:**
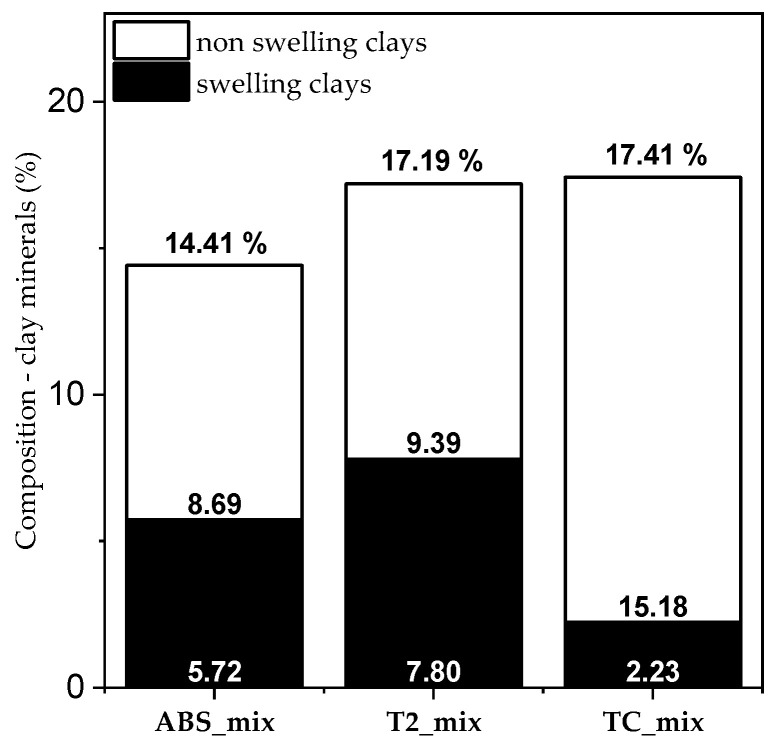
Comparison of plasters: difference in swelling and non-swelling clay mineral contents.

The observed main difference in the plasters is the relative content of swelling (smectite) and non-swelling (illite, kaolinite and chlorite) clay minerals. T2-mix contained the highest amount of swelling clay minerals, opposite to TC-mix, which contained mostly non-swelling clay minerals (Figure 1). 

### 3.1. Adhesion Resistance 

**Figure 2:**
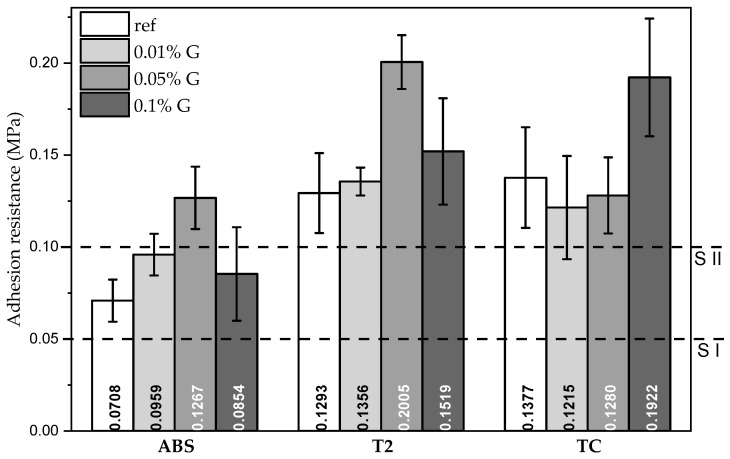
Adhesion resistance on standard hollow bricks for plasters.

The adhesion resistance values (MPa) on standard hollow bricks for each plaster are reported in Figure 2, along with the standard deviation of the values. All the mixtures attained the S-I class for adhesion resistance set by the DIN 18947 standard [30], overcoming the 50 kPa limit value. Class S-II (>100 kPa) was attained only by the T2 and TC plasters, even in their mixtures without additive; for ABS, instead, it was necessary to add 0.05 wt% graphene-based additive to attain this class. All plasters passed the test defined by the French Guidelines provided by AsTerre [24], which define the resistance threshold at 2 kg of load (7 kPa), which is lower than the weight of the container used in the experimental test. 

The different behavior of the soils was evidenced not only by the adhesion values of the reference mixes but also by the different change in properties when the additive was added. The ABS and T2 plasters followed a similar trend, with maximization of the adhesion with medium content of graphene-based additive, whereas for the TC plaster, it was necessary to add 0.1 wt% graphene-based additive content to achieve a significant improvement. 

### 3.2. Scanning Electron Microscopy 

**Figure 3:**
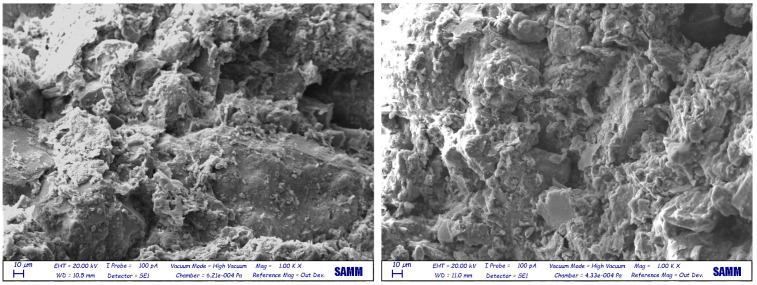
SEM images of TC and TC 0.1% G plasters fracture surfaces.

The morphology of plasters was also analyzed with a SEM through the SEI probe, which gives information about the surface morphology of the samples. The morphology change is not seen at this magnification either; see Figure 3.

**Figure 4:**
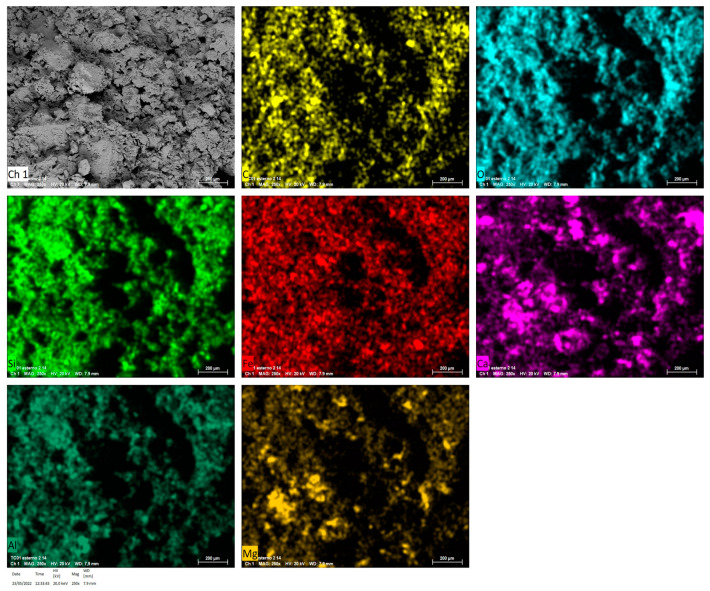
Composition map of TC 0.1% G sample.

Through EDS spectroscopy, instead, a compositional map can be created to confirm that graphene is homogeneously distributed within the sample. Figure 4 shows the distribution of the elements within the plaster mixture, from which the absence of graphene segregation can be confirmed. Here, the carbon distribution map follows exactly the distribution of the other elements that form the carbonates (O, Ca and Mg), leaving no region with only carbon, which would signal the presence of only graphene.

### 3.3. Capillary Rise

**Figure 5:**
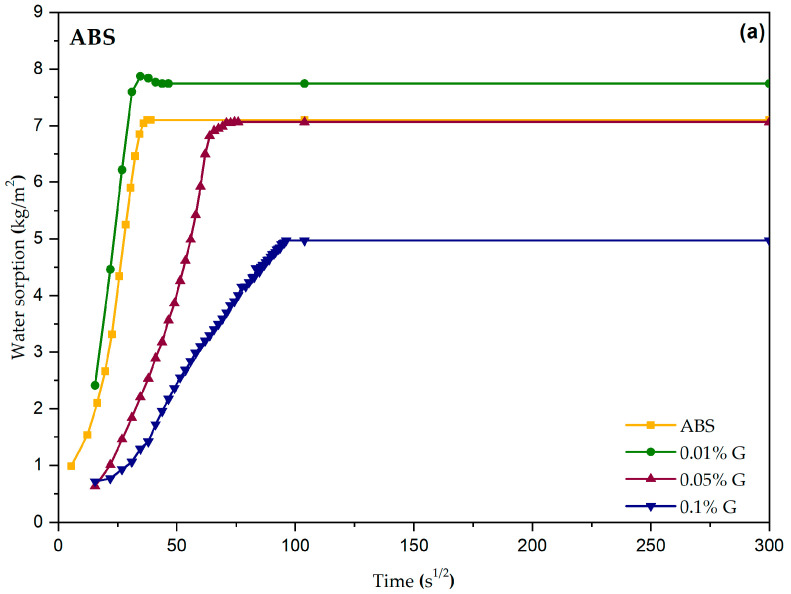

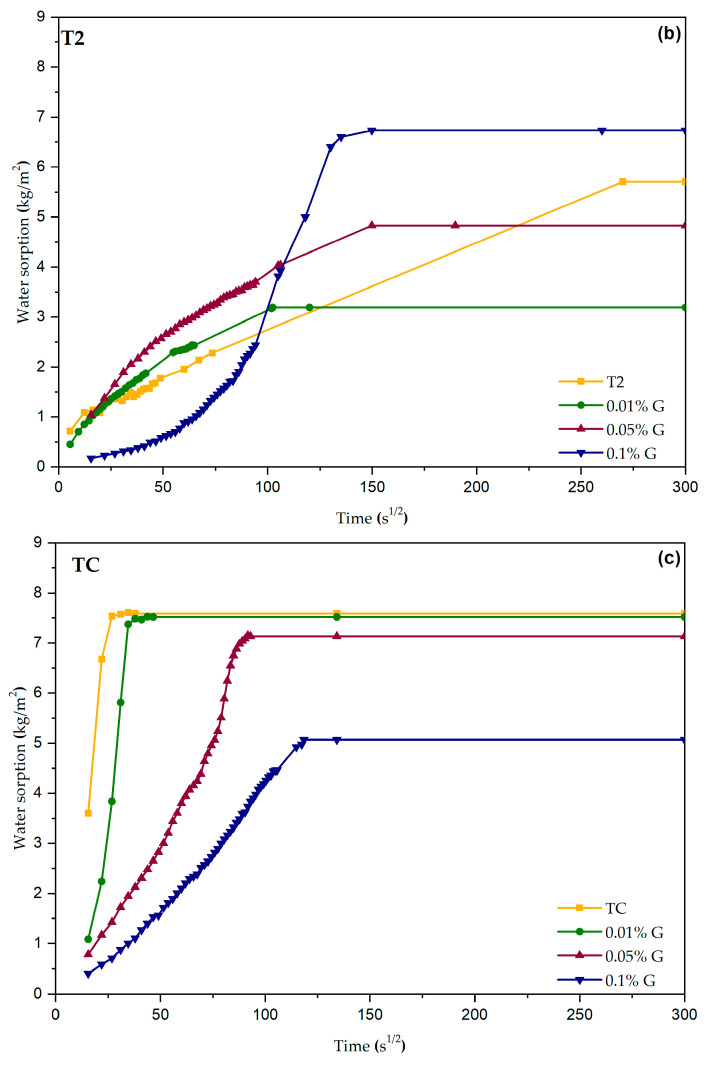
Sorption curves obtained in the capillary rise test for the plasters: (**a**) ABS, (**b**) T2 and (**c**) TC.

The sorption curves for all the plasters are shown in Figure 5. These curves should follow a linear trend for a short time and then move to a plateau value when saturation is reached. 

This behavior was found in the ABS and TC plasters (Figure 5a,c), whereas the T2 plaster (Figure 5b) strayed from this ideal trend, especially when looking at the one containing the highest graphene-based additive content. 

**Figure 6:**
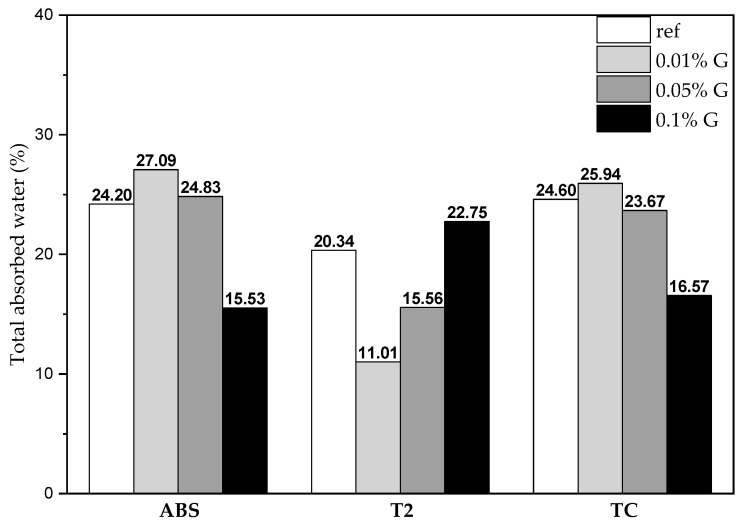
Total absorbed water in the capillary rise tests; values are expressed in w%.

The change in behavior for the T2 plasters is also seen in Figure 6, where it is evident that adding more graphene-based additive reduces the total absorbed water for ABS and TC (as is expected due to its hydrophobic nature), while it enhances water absorption in T2. 

### 3.4. Water Erosion Resistance

**Figure 7:**
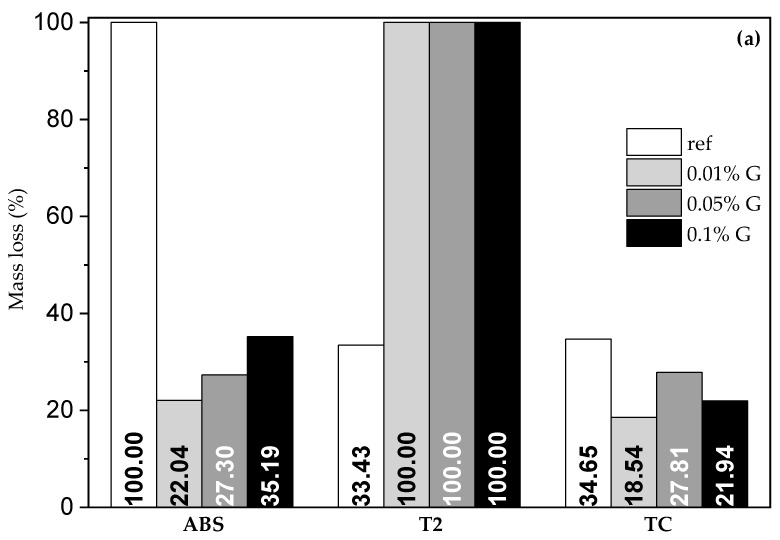

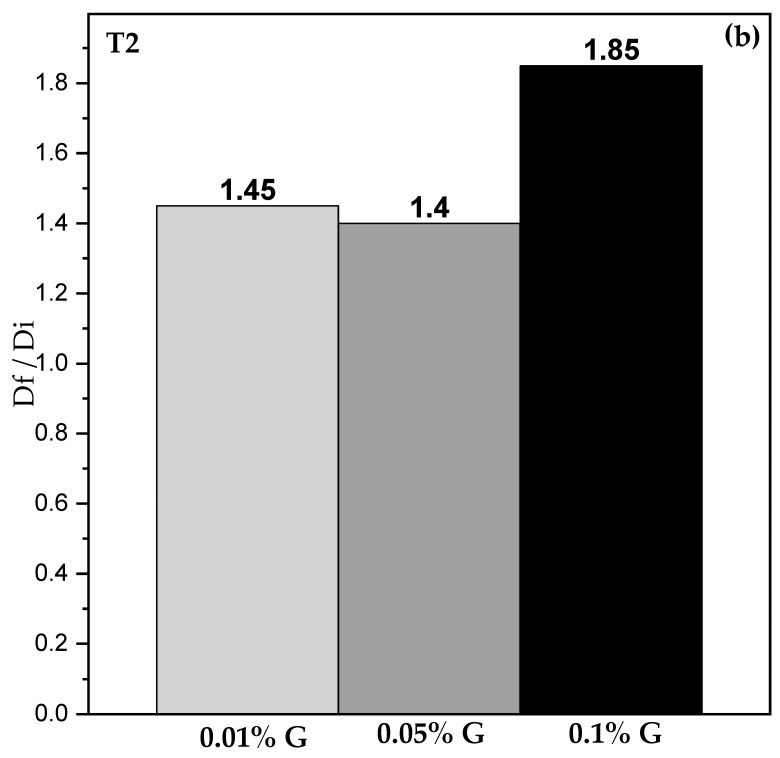
Drop test results: (**a**) mass lost in the 4 h test and (**b**) slump test for the T2 plasters with the graphene-based additive.

In the drop resistance test, it was observed that the graphene-based additive reacted differently with the three earth plasters: it improves both the water resistance of ABS and TC plasters at the lowest concentration (Figure 7a). The graphene-based additive had the opposite effect on the T2 plaster, which became completely fluid; in addition, the slump test on T2 samples showed that the more graphene-based additive was added, the higher the fluidity of the mixture was, producing a worsening effect (Figure 7b).

### 3.5. Earth Plasters’ Performance with Graphene-Based Additive

In order to better understand the nature of the interaction between the raw earths and the graphene-based additive and investigate whether its presence may change the microstructure of the clays, XRD and FTIR characterization were also run on the samples with graphene-based additive.

### 3.6. XRD 

**Figure 8:**
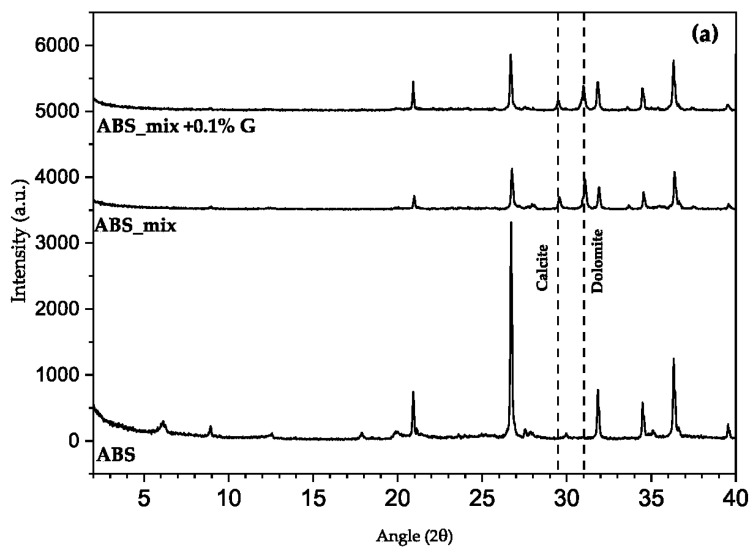

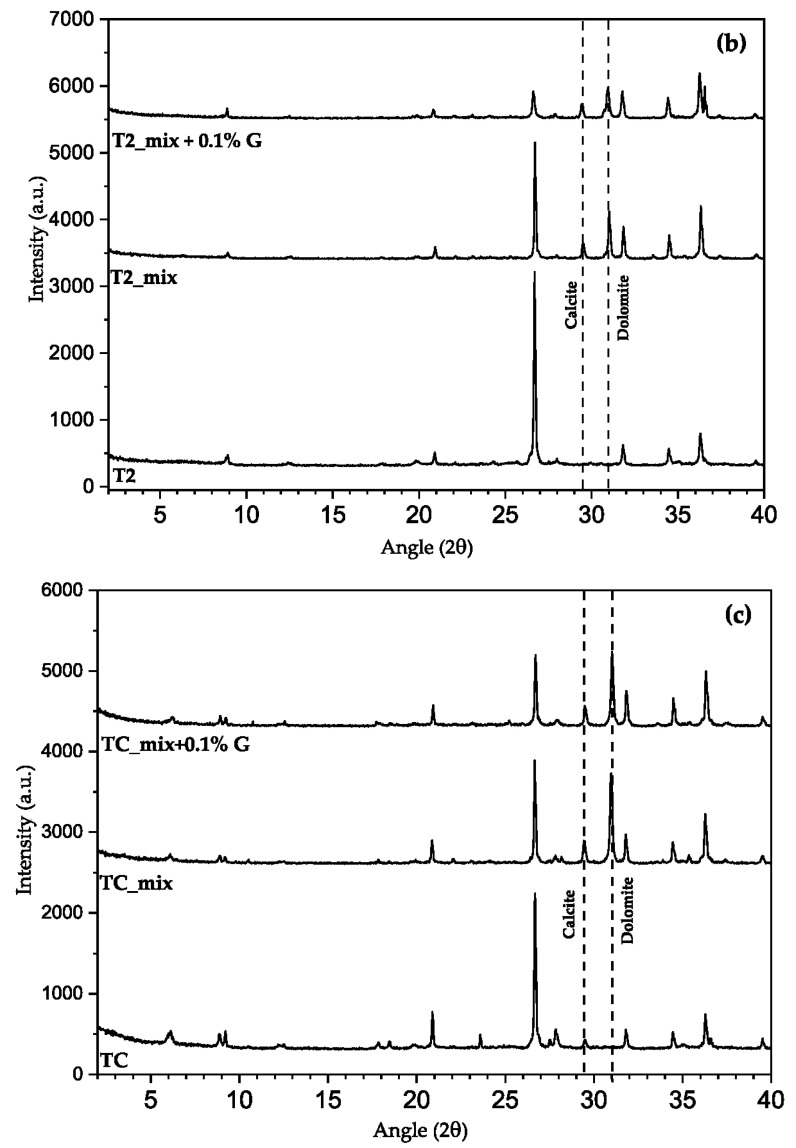
XRD patterns for (**a**) ABS, (**b**) T2 and (**c**) TC plasters.

In the XRD patterns of the plasters with the highest graphene-based additive content (0.1%), no peaks are changed, and no new peaks appear compared with the diffractogram of the reference plaster mix (Figure 8). The graphene-based additive is not visible in the data because its characteristic peak is obscured by the high amount of quartz at 2θ 26.6°.

### 3.7. ATR-FTIR

**Figure 9:**
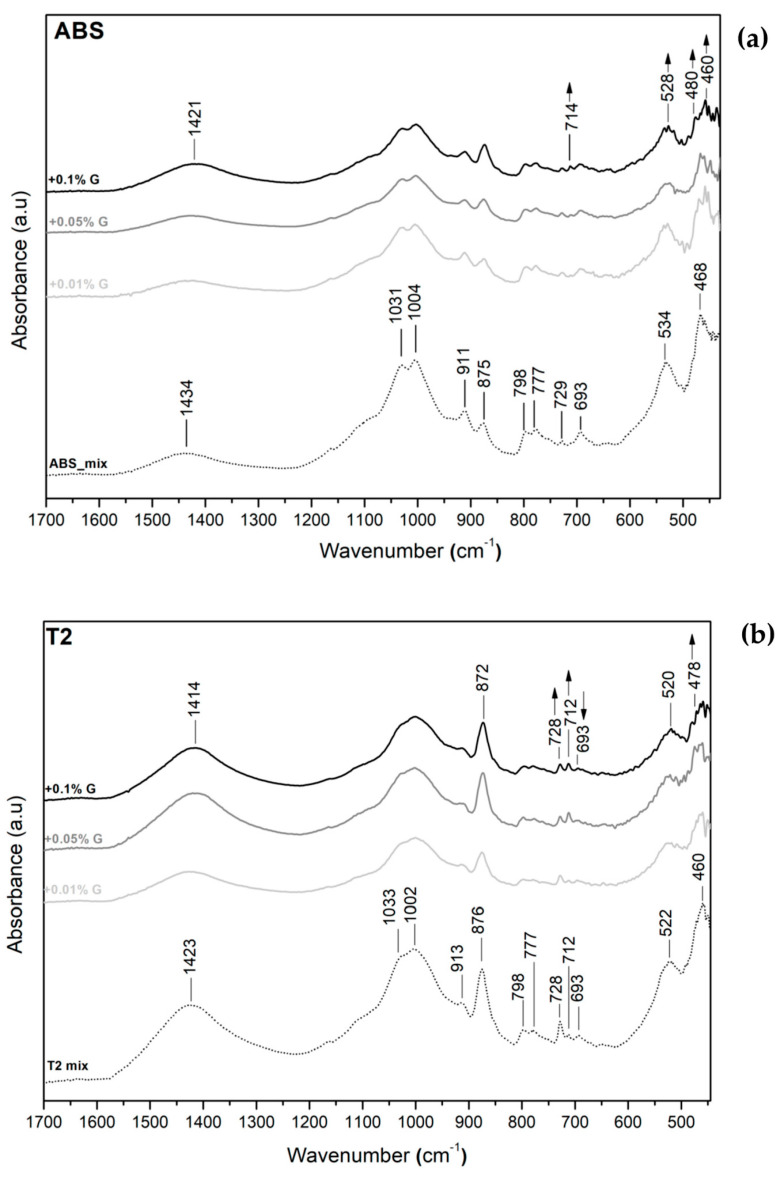

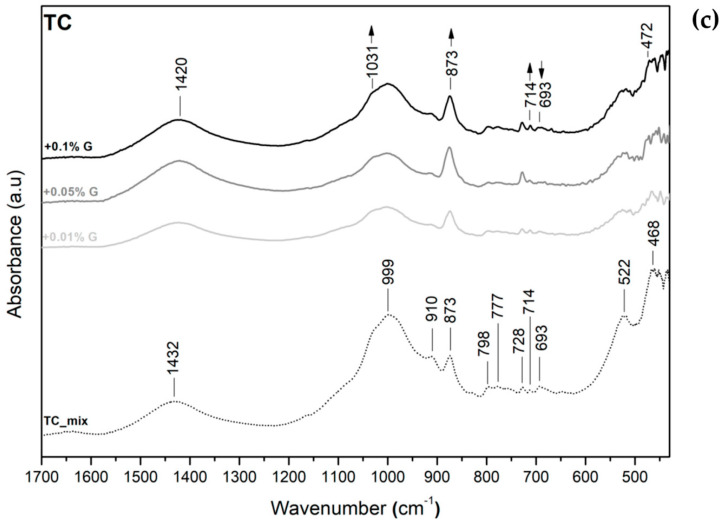
ATR-FTIR (**a**) ABS, (**b**) T2 and (**c**) TC plasters.

For all plasters, with the increase in the concentration of graphene-based additive, the spectra do not show new bands, because the content was too small compared with the quantity of minerals (Figure 9). 

The only visible change is the modification of the relative intensities of some bands as pointed out by the arrows. The peaks that are modified are those referring to the illite–smectite interstratified minerals in the SI-O bands (between 532 and 460 cm^−1^), as well as the 693 cm^−1^ peak in T2 and TC, which is due to illite [31]. Another visible change in the C-O stretching bands is related to the intensification of the calcite–dolomite peaks and the overall decrease in intensity of all other peaks. This is only an indication that the chemical environment around these minerals has slightly changed with the addition of the graphene-based additive.

### 3.8. Atterberg Limits

There was no clear trend for the influence of graphene-based additive on the liquid or plastic limit but the plasticity index decreased; this change was very small for TC, but it became a 50% reduction for T2 (Table 2 and Table 4). The reduction in the plasticity index (the difference between liquid and plastic limits) with the addition of the additive has also been reported in the literature (in the case of cement-stabilized soil with graphene additive) and is thought to be due to higher compaction [16], which could be correlated to the lubricant action of graphene, which could enhance soil grain arrangement during compaction to reach a denser state. 

## 4. Discussion

The comparison of the three reference mixtures shows that the ABS plaster performed poorly compared with the TC and T2 ones because of its lower clay mineral content, as it resulted from the XRD analysis [31] (Table 3). 

**Figure 10:**
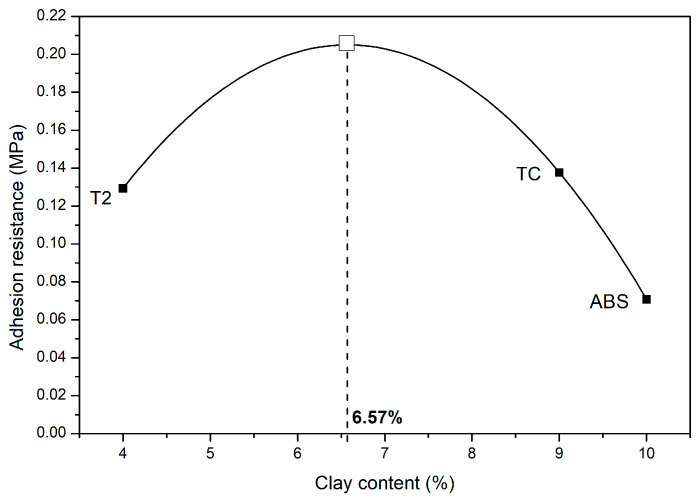
Optimal clay content to maximize adhesion on standard hollow bricks.

Furthermore, by looking at the cumulative particle size distributions of the mixes containing clay and sand, the clay contents of the ABS, T2 and TC mixes were 10%, 4% and 9%, respectively. When comparing the adhesion performance with these values, it is noticeable that the optimal content of clays to optimize adhesion on bricks is between 4% and 9% (Figure 10). This correlation has also been found in the literature, even though the value of optimal clay content is said to be strongly dependent on the type of clay, surface preparation and surface of deposition [32].

**Figure 11:**
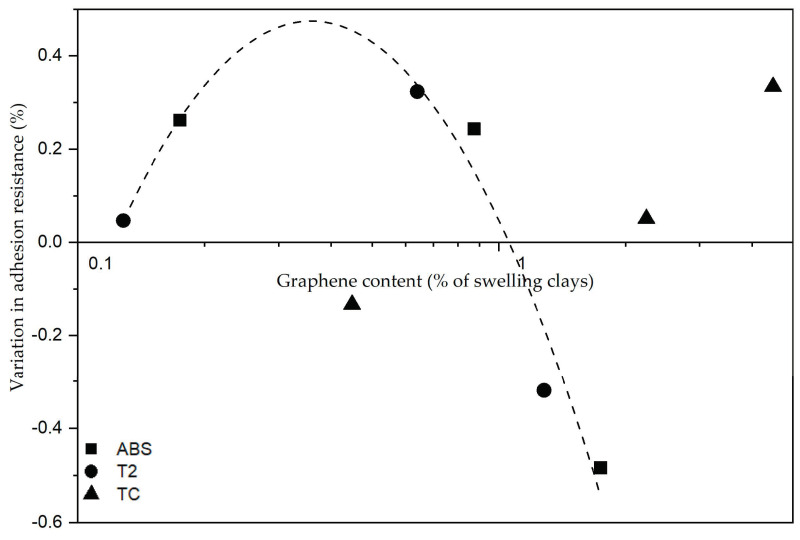
The change in adhesion behavior depending on the graphene-based additive content with respect to the swelling portion of the clays.

It was also observed that the adhesion performance depended on the percentage of graphene-based additive with respect to the swelling minerals and followed the same trend for the ABS and T2 plasters, while it was completely different for the TC samples (Figure 11). This suggests some kind of interaction with the swelling portion of the clay minerals, which cannot be so strongly seen in the TC plaster given its much lower swelling clay content.

The additive acts in different ways on the capillary rise test on the different plasters, as already mentioned, and this could be again related to a different swelling clay content.

The exponential-increase anomaly in water absorption shown by the T2 + 0.1% G plaster was also present (even though it was less visible) in the ABS and TC plasters with graphene-based additive. This change in behavior may be related to water absorption of the clay minerals [26], and it should be reduced when stabilizing additives are added to the plaster mixtures. This clearly shows that this graphene additive does not work as a stabilizer, hindering the swelling of smectite but enhancing the anomaly in water sorption.

Moreover, the peculiarity of the T2 plasters’ behavior in water resistance tests could also be related to the different earth-to-sand ratio; this difference entails a lower content of earth (keeping the volume of the sample constant), which could mean that the graphene separates the soil and sand portions, acting as a kind of lubricant, or that by interacting with the earth portion, it somehow reduces its binding properties. This hypothesis is consistent with experimental observations after the drop tests, where it seemed like the graphene in the T2 plasters separated the earth and sand fractions. For these tests, the greatest anomaly was found in the T2 plaster, and a drastic change in behavior was also visible in the tests, where this plaster with graphene quickly became fluid rather than obtaining higher resistance to water, as it happened for the other two. The effect of the graphene additive seems to be between the grains of sand and soil, where it can influence the interaction with water through its hydrophobicity. In other words, water preferentially gets absorbed in the interlayer region of the swelling clays instead of flowing in the pores between the grains of sand and soil. This could also explain the anomaly in capillary rise identified in the T2 plasters, whereby initially, water was absorbed by the swelling clays (slow capillary rise within the pores) and, after saturation, it started to fill the inter-particle pores, generating a visible rise in the level of water within the samples.

The evidence found in the ATR-FTIR spectra shows only a small change in the intensity of the illite (691 cm^−1^) and illite–smectite peaks (in the 560–480 cm^−1^ region), whilst XRD analysis did not provide further information about graphene–clay interaction.

By analyzing the different performance of the plasters in the experimental tests, it seems that the difference in behavior is determined by the presence of swelling clays. In adhesion tests, the ABS and T2 plasters showed the same behavior, which was different from that of the TC plasters, containing less swelling clays. The adhesion performance of the ABS and T2 plasters, with the addition of graphene additive, followed the same trend, whereas the TC plasters showed a peak at higher percentages, suggesting that the optimal graphene content would be higher for this series of plasters (Figure 11). This is possibly due to the compacting action of the graphene additive; such higher compaction is also supported by the lowering in plasticity of the T2 soil seen in the re-evaluation of the Atterberg limits.

## 5. Conclusions

As previously discussed, the use of earthen building materials has many advantages also in terms of sustainability [33]; in particular, earth-based mortars are today widely applied. Through the experimental tests carried out, in this paper, it has been possible to investigate the different performance of three earth plasters with graphene-based additive (0.01 wt%, 0.05 wt% and 0.1 wt% of the earth–sand solid portion). The plaster properties assessed in this study are adhesion strength, capillary rise, and rainfall water erosion resistance. 

It has been found that the graphene additive improves the adhesion performance of all plasters but the effect of the additive changes depending on the plaster: ABS and T2 show a similar trend, whereas TC-based plasters, which are richer in non-swelling clays, show an improvement in adhesion only with the highest graphene content. It has been hypothesized that the presence of graphene could help increase the cohesion of plasters by reducing the internal frictional angle, thereby improving adhesion performance. 

On the other hand, in the water interaction tests, i.e., capillary rise and erosion resistance, the hypothesized presence of the graphene additive in the voids between the earth and sand particles seems to be detrimental, because its hydrophobicity would determine enhanced absorption of water within the planes of swelling clays. The T2 samples, which are the ones with the highest swelling clay content, in fact, are the ones where the addition of graphene determines the strongest reduction in resistance towards water. 

## Figures and Tables

**Table 1:** The mineralogical composition of the raw earth, obtained through XRD analysis. The values are expressed in w%.

	Quartz	Calcite	Albite	Microcline	Illite	Chlorite	Smectite	Kaolinite	Amorphous
ABS	32.5	-	3.14	5.27	10.90	3.65	8.98	-	35.60
T2	36.9	-	6.19	4.94	15.94	4.79	9.80	5.38	16.06
TC	46.1	5.24	8.25	6.60	17.40	4.16	6.88	5.88	-

**Table 2:** Geotechnical parameters of the raw earths [29].

	Water Content (%*g*/*g*)	Specific Gravity (-)	Granulometric Parameters			Atterberg Limits		Shrinkage Limit (%)
			Clay Content (%)	Silt Content (%)	Sand Content (%)	Liquid Limit (%)	Plastic Limit (%)	
ABS	3.1	2.78	36	44	20	45	30	13.7
T2	3.5	2.77	21	70	9	54	15	9.5
TC	5.1	2.79	30	54	16	38	24	19.3

**Table 3:** Mineralogical composition of the plaster mixes, obtained through XRD analysis. The values are expressed in w%.

(%)	Quartz	Illite	Kaolinite	Chlorite	Smectite	Calcite	Dolomite	Feldspars	Amorph.
ABS-mix	13.57	6.51	-	2.18	5.72	5.07	19.20	5.89	41.86
T2-mix	18.38	5.89	1.5	2.00	7.80	6.75	13.33	5.73	38.80
TC-mix	21.04	9.80	2.9	2.48	2.23	7.87	23.75	10.55	19.40

**Table 4:** Atterberg limits evaluated for a mixture of earth and graphene additive.

	Liquid Limit	Plastic Limit	Plasticity Index
ABS + 0.1% G	40	25	15
T2 + 0.1% G	54	34	20
TC + 0.1% G	43	31	11

## Data Availability

All data are contained within the article.
